# Supplementary material for: NOMA-MIMO in 5G network: a detailed survey on enhancing data rate
Source: PeerJ Comput Sci. 2025 Jan 31;11:e2388. doi: 10.7717/peerj-cs.2388 (PMC11888882; doi:10.7717/peerj-cs.2388)
Supplement: Supplemental Information 1 [file peerj-cs-11-2388-s001.pdf]

Straight2Bank
PAYEE ADVICE

Standard Chartered

SCB Ref : MY11064R2351666
Customer Ref : 2000018598243201
Date : 08/11/2024

To: PEERJ, INC

US
/UNITED STATES

**Invoice Total**	**2,443.00**
- Benef. Charge	0.00
- Discount	0.00
UTR Reference	ST25962411080341

Dear Sir / Madam (s),

We have on 08/11/2024 made a payment to your account xxxxxxxx0870 at CHOICE FINANCIAL GROUP for USD 2,443.00 as instructed by UNIVERSITI TELEKOM SDN. BHD..

Should you not receive the payment in time, please contact UNIVERSITI TELEKOM SDN. BHD. for further investigations.

Remittance Advice

This section includes details as supplied by UNIVERSITI TELEKOM SDN. BHD.

Payment Details :

Reference	Date	Description	Amount (USD)
09132024-982	13/09/2024	09132024-98284 : 09132024-98284 PC/2024/166109 Mardeni	2,443.00

*Note: Payment is made at the direction of, and based on information provided by, the originator of the payment instruction. If incorrect information is given or the originator revokes the payment instruction or any other circumstances arises which is beyond our control, this may result in payment being delayed or not received. The actual date of credit funds is also dependent on the clearing system(s) used and the beneficiary's bank.

Straight2Bank
MT103 PAYEE ADVICE

ScbRef: MY11064R2351666
CustRef: 2000018598243201
Date: 08/11/2024 11:05

Dear Sir / Madam (s),

Please be advised that the following payment has been made to your account on behalf of

UNIVERSITI TELEKOM SDN. BHD.

Payment Details

20	ST25962411080341
23B	CRED
32A	241108 USD 2443,00
33B	USD 2443,00
36	0,000000
50K	UNIVERSITI TELEKOM SDN. BHD.<br>TRAINING ROOM NO.2,LEVEL 2,TM<br>CONVERSATION CENTER,50672,JALAN<br>PANTAI BAHARU, KUALA LUMPUR.
57A	CHFGUS44021
59	/xxxxxxxx0870<br>PEERJ, INC<br>US<br>/UNITED STATES
71A	OUR
71F	0,00
71G	0,00

*Note: Payment is made at the direction of, and based on information provided by, the originator of the payment instruction. If incorrect information is given or the originator revokes the payment instruction or any other circumstances arises which is beyond our control, this may result in payment being delayed or not received. The actual date of credit funds is also dependent on the clearing system(s) used and the beneficiary's bank.
